# Inactivation of the three *GGA* genes in HeLa cells partially compromises lysosomal enzyme sorting

**DOI:** 10.1002/2211-5463.13040

**Published:** 2021-01-01

**Authors:** Balraj Doray, Lin Liu, Wang‐Sik Lee, Benjamin C. Jennings, Stuart Kornfeld

**Affiliations:** ^1^ Department of Internal Medicine Washington University School of Medicine St. Louis MO USA; ^2^ Present address: M6P Therapeutics 20 S. Sarah Street St. Louis MO 63108 USA

**Keywords:** AP‐1, cathepsin D sorting, CI‐MPR, GGA1, GGA2, GGA3

## Abstract

The Golgi‐localized, gamma‐ear containing, ADP‐ribosylation factor‐binding proteins (GGAs 1, 2, and 3) are multidomain proteins that bind mannose 6‐phosphate receptors (MPRs) at the Golgi and play a role, along with adaptor protein complex 1 (AP‐1), in the sorting of newly synthesized lysosomal hydrolases to the endolysosomal system. However, the relative importance of the two types of coat proteins in this process is still unclear. Here, we report that inactivation of all three *GGA* genes in HeLa cells decreased the sorting efficiency of cathepsin D from 97% to 73% relative to wild‐type, with marked redistribution of the cation‐independent MPR from peripheral punctae to the *trans*‐Golgi network. In comparison, *GNPTAB^−/−^* HeLa cells with complete inactivation of the mannose 6‐phosphate pathway sorted only 20% of the cathepsin D. We conclude that the residual sorting of cathepsin D in the GGA triple‐knockout cells is mediated by AP‐1.

## Graphical abstract

In this study, we inactivated all three GGA genes in HeLa cells using CRISPR‐Cas9 genome editing. The GGA triple‐knockout cells secreted 27% of newly synthesized cathepsin D, compared to 2.6% secretion seen with wild‐type HeLa cells. In contrast, HeLa cells with complete inactivation of the mannose 6‐phosphate pathway secreted 77.5% of the newly synthesized cathepsin D.

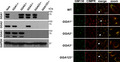

**Abbreviations**
GGAs: Golgi‐localized, gamma‐ear containing, ADP‐ribosylation factor‐binding proteins
AP‐1: adaptor protein complex 1
MPRs: mannose 6‐phosphate receptors
CI‐ & CD‐MPR: cation‐independent and cation‐dependent MPR
M‐6‐P: mannose 6‐phosphate

A critical step in the trafficking of newly synthesized acid hydrolases to lysosomes occurs at the *trans*‐Golgi network (TGN) where mannose 6‐phosphate receptors (MPRs) bind the acid hydrolases via their mannose 6‐phosphate (M‐6‐P) tags. The receptor–ligand complexes are then incorporated into clathrin‐coated vesicles (CCVs), followed by transport to the endosome/lysosome compartment [1].

Key to the assembly of the CCVs are two coat proteins, the Golgi‐localized, gamma‐ear containing, ADP‐ribosylation factor‐binding proteins (GGAs) and adaptor protein complex 1 (AP‐1), both of which bind clathrin and the MPRs [2, 3]. A number of studies in mammalian cells have used RNA‐interference (RNA‐i) to knock down the various GGAs to determine the role of each GGA in acid hydrolase trafficking to lysosomes. These studies have indicated that all three GGAs play a role in this process [4, 5, 6]. In addition, triple‐knockdown of the GGAs reduced the sorting efficiency of the lysosomal enzyme, cathepsin D, from 80% in control cells compared to 68% in the cells treated with siRNA targeting the three GGAs [6]. Presumably, the residual sorting is mediated by AP‐1. A limitation of the RNA‐i experiments is that the knockdowns were not complete with 5–10% of each GGA remaining. Consequently, it has not been possible to accurately assess the requirement for the GGAs in acid hydrolase sorting.

To address this issue, we generated HeLa cells in which all three *GGA* genes have been inactivated using CRISPR‐Cas9 genome editing. These cells have no detectable GGAs. The effect on the sorting of cathepsin D and several other lysosomal acid hydrolases was examined to determine the consequence of the total loss of GGAs on lysosomal enzyme trafficking.

## Materials and methods

### Cell lines

The parental WT and various GGA knockout HeLa cell lines (see below) were maintained in Dulbecco's Modified Eagle Medium (DMEM) (Life Technologies, Carlsbad, CA, USA) containing 0.11 g·L^−1^ sodium pyruvate and 4.5 g·L^−1^ glucose, supplemented with 10% (vol/vol) FBS (Atlanta Biologicals, Flowery Branch, GA, USA), 100 000 U·L^−1^ penicillin, 100 mg·L^−1^ streptomycin (Life Technologies), and 2 mm l‐glutamine (Life Technologies). The generation of *GNPTAB^−/−^* HeLa cells has been described in detail [7], and these cells were similarly maintained in DMEM.

### Generation of GGA knockout cell lines by CRISPR Cas9 genome editing

GGA knockout cell lines were generated using the plasmid pX330‐U6‐Chimeric_BB‐CBh‐hSpCase9 (Addgene no. 42230). All sgRNAs were designed by E‐CRIPSR and CRISPR‐Era online software. The following sgRNAs (NGG protospacer adjacent motif shown in lowercase) were used for generating the various cell lines: *hGGA1* sgRNA with the sequence ^5′^GAAACATGCATGAAGAGCTGcgg^3′^ (made by annealing GGA1 oligonucleotides ^5′^caccGAAACATGCATGAAGAGCTG^3′^and ^5′^aaacCAGCTCTTCATGCATGTTTC^3′^); *hGGA2* sgRNA with the sequence ^5′^GGAAGCTCTTTATGCCTTAAcgg^3′^ (made by annealing GGA2 oligonucleotides ^5′^caccGGAAGCTCTTTATGCCTTAA^3′^ and ^5′^aaacTTAAGGCATAAAGAGCTTCC^3′^); *hGGA3* sgRNA with the sequence ^5′^GGAGATTTCATAACGAAGTGggg^3′^ (made by annealing GGA3 oligonucleotides ^5′^caccGGAGATTTCATAACGAAGTG^3′^ and ^5′^aaacCACTTCGTTATGAAATCTCC^3′^). Following digestion of the vector pX330 vector with the restriction enzyme Bbs1, the three different *GGA* guide RNA plasmids were made by annealing the corresponding oligonucleotides and ligation into the cut vector. Transformants were screened by colony PCR and positive clones verified by Sanger sequencing (GENEWIZ, South Plainfield, NJ, USA). Parental HeLa cells were transfected with 500 ng pX330‐GGA plasmid and 150 ng pEGFP‐puro (Addgene no. 45561) in 12‐well plates with 50% confluence using Lipofectamine 3000 (Life Technologies) according to the manufacturer's protocol. Puromycin, at a concentration of 10 μg·mL^−1^, was added to the cell media the following day. Twenty‐four hours later, the cells were washed twice with PBS, trypsinized and counted, then diluted and plated to two to three 96‐well plates with approximately one cell per well. Cells in the 96‐well plate were cultured in 15% FBS for ~ 10 days. Single colonies were expanded and screened by western blotting using the anti‐GGA antibodies described below.

### Antibodies

The following published/validated commercial antibodies against human GGA proteins were used to screen for the GGA knockout cell lines: anti‐GGA1 rabbit monoclonal (Abcam, Cat # ab170956, Cambridge, MA, USA), anti‐GGA2 mouse monoclonal (BD Biosciences, Cat # 612612), and anti‐GGA3 rabbit monoclonal (Cell Signaling, Cat # D66F1, Beverly, MA, USA). The anti‐GM130 mouse monoclonal antibody was from BD Biosciences (Cat # 610822). The anti‐CI‐MPR rabbit polyclonal antibody was generated in our laboratory using soluble CI‐MPR purified from FBS [8], as was the anti‐cathepsin D rabbit polyclonal antibody.

### Immunoblotting

Proteins resolved by using SDS/PAGE under reducing conditions were transferred to nitrocellulose membrane and detected with antibodies as described in the figure legends. Equal amounts of whole‐cell extract were loaded on the gels.

### Cathepsin D sorting assay

HeLa cells (WT and knockout cells) at 90% confluency in 6‐well plates were labeled for 1 h with 1.07 mL cysteine/methionine‐free DMEM containing 700 μCi of TRAN 35S‐LABEL (conc. 10.5 mCi·mL^−1^; MP Biomedicals, Inc., Irvine, CA, USA) in the presence of 20 mm HEPES buffer and 10% dialyzed fetal calf serum. Excess cold methionine (2 mm final concentration) was added to initiate a 4‐h chase. Cathepsin D in the cells and media was immunoprecipitated with the rabbit polyclonal anti‐cathepsin D antibody and resolved by 10% SDS/PAGE under non‐reducing conditions. Gels were treated with EN^3^HANCE (PerkinElmer Life Sciences, Downers Grove, IL, USA), dried, and exposed to Kodak X‐Omat AR film at −70 °C. The resultant autoradiogram was used as a template to excise from the gel the regions corresponding to the processed and secreted forms of cathepsin D. The gel slices were then soaked in scintillation fluid overnight, and the associated radioactivity was determined on a Beckman model LS 6000SE liquid scintillation counter. The percentage of cathepsin D sorted was calculated as the ratio of radioactivity in the processed form divided by the sum of the processed and secreted forms.

### Lysosomal enzyme activity assay

Lysosomal enzyme activity assays were performed as described previously [9]. Briefly, β‐hexosaminidase (β‐Hex) and β‐galactosidase (β‐Gal) were assayed with 5 mm 4‐methylumbelliferyl(MU)‐N‐acetyl‐β‐d‐glucosaminide (Sigma‐Aldrich, St Louis, MO, USA) and 5 mm 4‐MU‐β‐d‐galactopyranoside (Calbiochem, San Diego, CA, USA), respectively, in 50 mm citrate buffer containing 0.5% Triton X‐100 (pH 4.5). The activity of α‐galactosidase (α‐Gal) was assayed with 5 mm 4‐MU‐alpha‐d‐galactopyranoside (Sigma‐Aldrich) in 50 mm citrate buffer containing 0.5% Triton X‐100 (pH 4.5). The activity of β‐glucuronidase (GusB) was assayed with 5 mm 4‐MU‐β‐d‐glucuronide (Calbiochem) in 0.1 m Na‐acetate buffer containing 0.5% Triton X‐100 (pH 4.6). The activity of β‐mannosidase (β‐Man) was assayed with 5 mm 4‐MU‐β‐d‐mannopyranoside (Sigma‐Aldrich) in 50 mm Na‐citrate buffer containing 0.5% Triton X‐100 (pH 5.0). Assay mixtures containing 40 μL of 1 mm substrate solution (final substrate concentration) and 10 μL of cell medium were incubated for 1–5 h at 37 °C and then quenched with 950 μL of 0.4 m glycine‐NaOH buffer, pH 10.8. The fluorescence was measured using a TURNER Model 450 Fluorometer (Barnstead Thermolyne Corporation, Dubuque, IA), with excitation and emission wave lengths of 360 and 450 nm, respectively.

### Immunofluorescence microscopy

To determine the subcellular localization of CI‐MPR, WT or GGA knockout cells were plated overnight on sterile glass coverslips, fixed the following morning with 4% formaldehyde (Sigma‐Aldrich) for 10 min, permeabilized and blocked with PBS containing 0.4% (v/v) Triton X‐100% and 2% immunoglobulinG‐free BSA (Jackson ImmunoResearch, West Grove, PA, USA) for 1 h, and then probed with the indicated combinations of primary antibodies in PBS containing 0.1% Triton X‐100% and 0.5% BSA. Following fluorophore‐conjugated secondary antibody treatment and washing, the processed cells were mounted in ProLong® Gold antifade mounting medium (Life Technologies), and the images were acquired with an LSM880 confocal microscope (Carl Zeiss Inc., Peabody, MA, USA). Images were analyzed by imagej software (NIH, Bethesda, MD, USA).

## Results

### Inactivation of *GGA* genes in HeLa cells using CRISP‐Cas9 genome editing

**Fig. 1:**
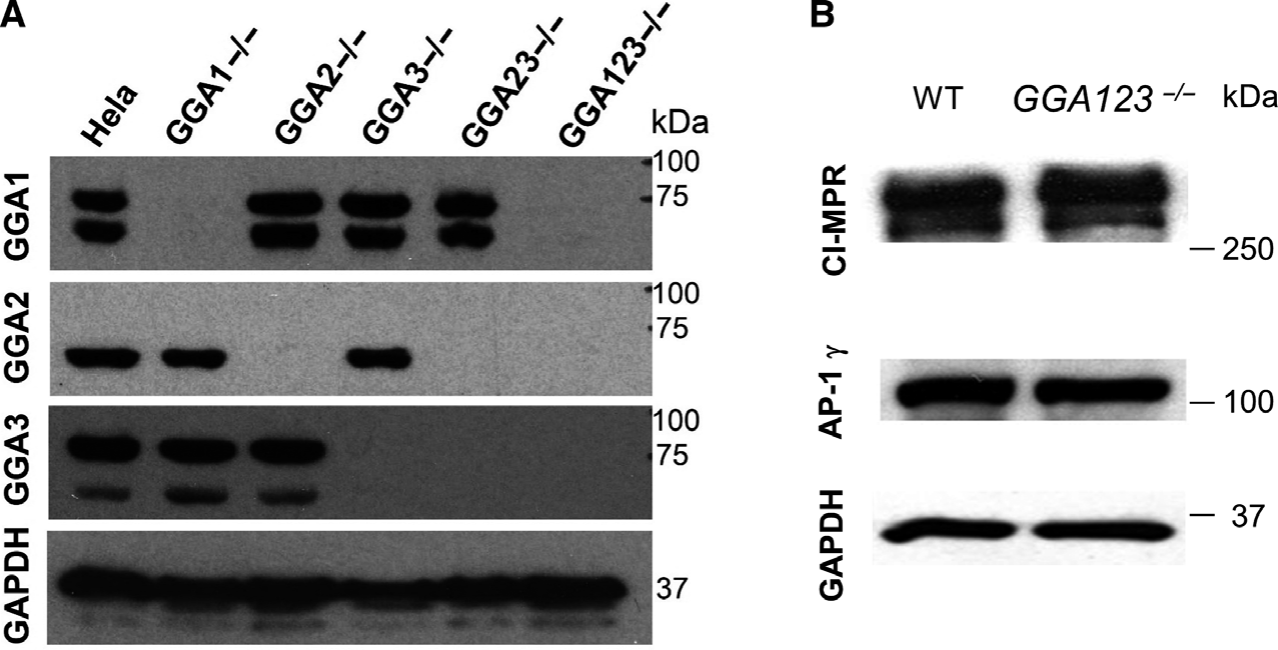
(A) Immunoblot analysis of endogenous GGA expression in the indicated cell lines, as determined by probing with the various anti‐GGA antibodies. (B) Expression of CI‐MPR and AP‐1 in parental versus *GGA123^−/−^* triple‐knockout HeLa cells, as determined by immunoblot analysis.

Since HeLa cells express all three *GGA* genes, we first inactivated each gene individually using the vector pX330, with the corresponding guide RNA sequence. Starting with *GGA2^−/−^* cells, the *GGA123^−/−^* triple‐knockout HeLa cells were made by sequentially inactivating the *GGA3* gene, followed by the *GGA1* gene. Immunoblots of the parental wild‐type (WT) HeLa cells and the 5 generated knockout cell lines, using reliable anti‐GGA antibodies, show that except for the control WT HeLa cells, there is no detectable expression of the targeted GGAs in their respective cell lines (Fig. 1A). For the GGA1 and GGA3 immunoblot blots, a faster migrating lower band was also observed in addition to the upper band corresponding to the full‐length protein. Since the lower band disappeared upon inactivation of the respective *GGA* gene, it is likely a degradation product. We did not observe a change in the level of expression of either AP‐1 or CI‐MPR between WT and the *GGA123^−/−^* HeLa cells (Fig. 1B), or the single GGA knockout HeLa cells (not shown). For the rest of this study, we chose to analyze only the individual and triple‐knockout cells.

### Cathepsin D sorting is impaired in *GGA2^−/−^* and *GGA123^−/−^* HeLa cells

**Fig. 2:**
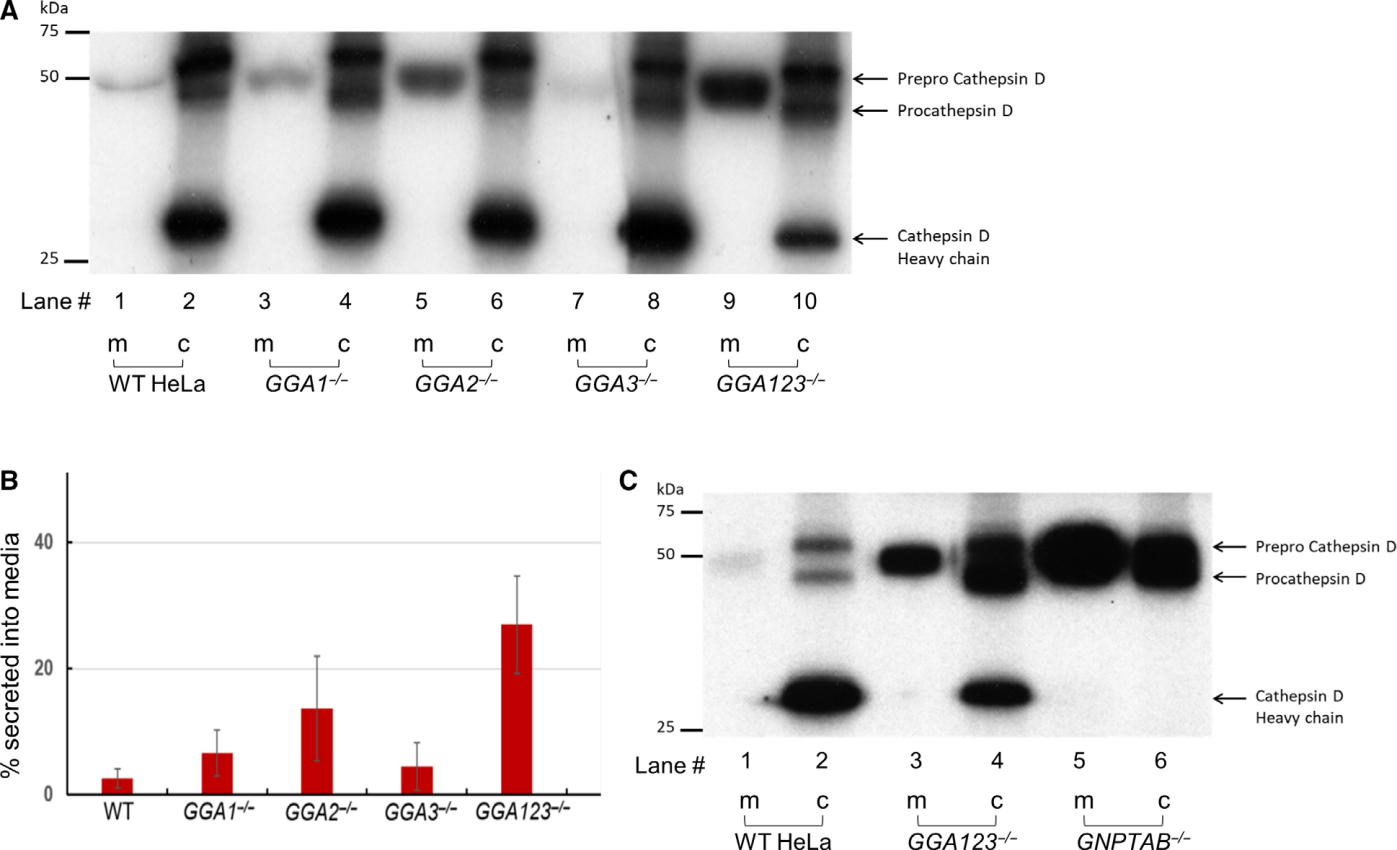
Sorting of cathepsin D in the absence of GGA proteins. (A) Parental WT, *GGA1^−/−^*, *GGA2^−/−^*, *GGA3^−/−^*, and *GGA123^−/−^* HeLa cell lines were metabolically labeled with [^35^S]methionine/cysteine and processed as described under ‘Materials and methods’. Secreted and intracellular cathepsin D molecules were immunoprecipitated with a polyclonal anti‐cathepsin D antibody, resolved by 10% SDS/PAGE and visualized by autoradiography of the dried gel. A representative autoradiograph is shown. (B) The percentage of cathepsin D in the media (m) was calculated as the ratio of radioactivity in the secreted form divided by the sum of the processed (c) and secreted forms (m). The data shown are the mean ± SD for four independent experiments. (C) Parental WT, *GGA123^−/−^*, and *GNPTAB^−/−^* HeLa cell lines were processed for cathepsin D sorting as described in (A). A representative autoradiograph is shown, and data presented are the mean of two independent experiments.

Several studies have looked at the impact of RNA‐i of GGAs, either individually or in combination, on the sorting of the lysosomal enzyme cathepsin D [4, 5, 6]. In these studies, knockdowns of > 90% efficiency were routinely achieved. However, the presence of three homologous GGAs with partly overlapping functions means that even a 5% residual activity of each GGA in the triple‐knockdown cells could have a cumulative effect. Our triple‐knockout cell line allows for the assessment of endogenous cathepsin D sorting in the complete absence of GGAs, following cellular adaptation in the presence of only AP‐1. Pulse‐chase experiments, followed by immunoprecipitation with anti‐cathepsin D anti‐sera, revealed no significant difference in the amount of secreted cathepsin D, as determined from the radioactive counts in the media (m) as a fraction of the total processed (c) and secreted forms (m), between the WT (2.6 ± 1.5%), *GGA1^−/−^* (6.7 ± 3.7%), and *GGA3^−/−^* (4.5 ± 3.8%) HeLa cells (Fig. 2A, lanes 1, 3, and 7, and Fig. 2B), since the majority of the cathepsin D was properly transported to lysosomes where it was cleaved to the mature form (Fig. 2A, lanes 2, 4, and 8). In the case of the *GGA2^−/−^* cells, a significant, but modest, elevation (13.7 ± 8.2%) in cathepsin D secretion was noted (Fig. 2A, lane 5, and Fig. 2B). Cathepsin D secretion into the medium by the *GGA123^−/−^* triple‐knockout cells, on the other hand, was markedly elevated (27 ± 7.7%), with a concomitant decrease in the amount of mature cathepsin D (Fig. 2A, lanes 9 and 10, and Fig. 2B). In separate experiments, we sought to determine how cathepsin D sorting compared between the *GGA123^−/−^* HeLa cells and *GNPTAB ^−/−^* HeLa cells where the Man‐6‐P trafficking pathway is completely disrupted [7]. Measurement of the radioactive counts associated with the bands in the media (m), as a fraction of the total processed (c) and secreted forms (m), showed that the *GNPTAB^−/−^* cells were severely compromised in their ability to sort cathepsin D (Fig. 2C, lane 5), in which case 77.5% of the cathepsin D was secreted into the medium, with additional newly synthesized cathepsin D still progressing through the secretory pathway on the way out. In the case of the parental WT and *GGA123^−/−^* HeLa cells, 7.05% and 35.5%, respectively, of the cathepsin D were secreted into the media in these experiments. The absence of mature cathepsin D (heavy chain, Fig. 2C, lane 5) is due to a lack of intracellular cathepsin B in the *GNPTAB^−/−^* HeLa cells (our unpublished data), since cathepsin B mediates the conversion of pro‐cathepsin D to mature cathepsin D [10]. It should also be noted that the expression of cathepsin D is dramatically upregulated in *GNPTAB^−/−^* HeLa cells, at both the mRNA and protein level (our unpublished data).

### **Missorting of other lysosomal enzymes in *GGA123*** *^−/−^* **Hela cells**

In addition to analyzing trafficking of cathepsin D to the lysosomes, we measured the sorting of five other lysosomal enzymes. In these experiments, media from control, *GGA123^−/−^* and *GNPTAB^−/−^* HeLa cells were collected after growing the cells for 24 h in the presence of 10 mm M‐6‐P. The M‐6‐P was added to prevent reuptake of secreted enzymes. As shown in Table 1, the activities of β‐Hex, β‐Man, β‐Gal and α‐Gal in the media of *GGA123^−/−^* HeLa cells ranged from 9% (β‐Man) to 127% (β‐Gal) more than that observed with WT cells, depending on the enzyme. The activity of GusB, on the other hand, was indistinguishable from WT. As expected in the absence of the M‐6‐P trafficking pathway, the activities of all five enzymes in the media were markedly higher with the *GNPTAB^−/−^* HeLa cells relative to WT, ranging from a 33% increase for β‐Man to 1628% increase for β‐Gal (Table 1).

**Table 1:** Lysosomal enzyme activity in the media of HeLa cell lines. Data are the mean ± SEM of three independent experiments. Activity is expressed as the percentage of enzyme in the medium to the total activity in the medium and cells.

	Gus B	β‐Hex	β ‐Man	β‐Gal	α‐Gal
*WT*	15.1 ± 2.0	33.9 ± 5.2	66.8 ± 9.3	4.0 ± 1.2	24.4 ± 7.5
*GGA 123 ^−/−^*	13.6 ± 2.6	52.1 ± 6.4	72.7 ± 2.2	9.1 ± 3.6	38.9 ± 4.5
*GNPTAB ^−/−^*	30.8 ± 2.5	83.3 ± 2.5	88.5 ± 3.2	69.1 ± 6.4	69.6 ± 4.8

### Ablation of the three *GGA* genes redistributes the CI‐MPR from peripheral punctae to the TGN

**Fig. 3:**
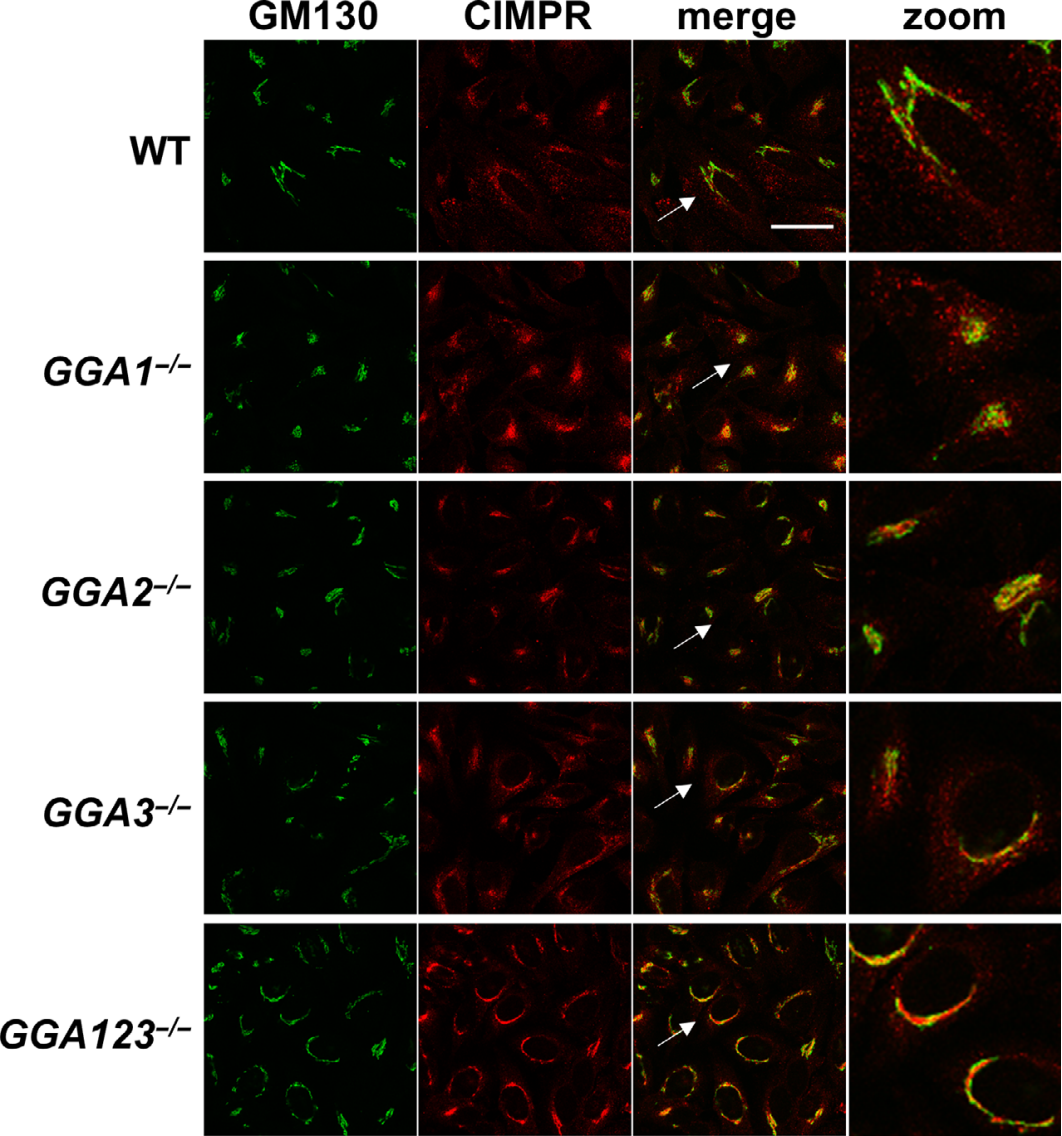
Cellular distribution of CI‐MPR in WT HeLa cells and the various *GGA* null cell lines, as determined by confocal immunofluorescence microscopy. Cells were co‐stained for the Golgi marker GM130. Scale bar: 40 µm. Zoom image is 3‐fold magnification of the region pointed by the arrow.

Since the GGAs bind directly to the cytosolic tails of the MPRs to facilitate packaging of the latter and their bound lysosomal enzymes into CCVs, our finding that cathepsin D and several other lysosomal enzymes are missorted in the absence of GGAs raised the possibility that the subcellular localization of the CI‐MPR might be impacted. To test this, confocal immunofluorescence microscopy was performed with the WT and the various *GGA* cell lines. The distribution of the CI‐MPR in WT cells showed a typical perinuclear pattern that partially overlapped with the Golgi marker, GM130, in addition to numerous peripheral punctae that did not colocalize with GM130 (Fig. 3). The CI‐MPR signal in the *GGA1^−/−^* and *GGA3^−/−^* cells showed a similar distribution, while the *GGA2^−/−^* cells displayed reduced peripheral punctae. The *GGA123^−/−^* triple‐knockout cells, on the other hand, showed an intense Golgi CI‐MPR signal that overlapped to a large extent with GM130, with a dramatic decrease in the number of peripheral punctae. It is of note that the GM130 signal in the triple‐knockout cells also showed a somewhat broader than normal Golgi staining, although the basis for this observation is not clear at the present time.

## Discussion

Since their discovery in 2000, one of the best characterized functions of GGAs is in the anterograde trafficking of the MPRs from the TGN to the endosomes [11, 12, 13]. It has been proposed by our group that the GGAs may co‐operate with AP‐1 to facilitate packaging of the MPRs, at least into a subpopulation of CCVs [14]. This hypothesis is supported by three lines of evidence: firstly, an interaction of GGAs with the γ‐appendage domain of AP‐1 [15, 16]; secondly, the presence of both mammalian GGA2 and AP‐1, or *Drosophila* GGA and AP‐1, within the same CCVs in a subpopulation of these vesicles, that were observed in thin cryosections of mouse L cells or *Drosophila* Dmel2 cells subjected to electron microscopy (EM) [6, 14]; and finally, the dependence of GGA2 on AP‐1 for incorporation of the former into CCVs [17]. These data are not mutually exclusive with the idea that GGAs and AP‐1 are able to nucleate their own CCVs, with each CCV‐type transporting a specific but overlapping complement of receptors and cargo.

The cathepsin D sorting data presented here clearly demonstrate that the trafficking of this enzyme to the lysosome is impaired by the simultaneous inactivation of the three *GGA* genes. At the same time, the deficiency is not as severe as with inactivation of the *GNPTAB* gene [7], which results in a complete blockade of the M‐6‐P‐dependent trafficking pathway. This shows that AP‐1, presumably by itself, is able to correctly sort a significant fraction of the cathepsin D bound to the MPRs. Our assays of the five different lysosomal enzymes secreted into the media show that with the exception of GusB, which was essentially the same between WT and the *GGA123^−/−^* triple‐knockout cells, the remaining four enzymes had elevated activity in the media in the absence of GGAs. One potential explanation for this is that GusB, and perhaps a few other lysosomal hydrolases, are segregated at the TGN into the nucleating CCVs containing AP‐1 only, while other lysosomal hydrolases are concentrated into GGA‐only CCVs, and some into CCVs containing both AP‐1 and GGA2 [14, 17]. The CI‐MPR and cation‐dependent MPR (CD‐MPR) together sort almost the complete repertoire of acid hydrolases from the TGN to the endosomes [18] While there is overlap between the two MPRs in the sorting of some acid hydrolases, others are sorted predominantly by either the CI‐MPR or CD‐MPR [19]. The fact that the two MPRs have quite different affinities for the GGAs [13, 20] lends support to the idea that, perhaps, some acid hydrolases are transported independent of the GGAs in AP‐1‐only CCVs.

Over the last two decades, numerous publications of the GGA proteins in tissue culture cells have identified a myriad of binding partners and functions for these monomeric clathrin adaptors [2]. Employing siRNA targeting the individual GGAs, some studies have attributed distinct roles for each GGA [21, 22, 23], while other studies have found overlapping roles [24, 25], or even antagonizing roles [26], depending on the pathway that was being studied. In addition, our analysis of *Gga* null mice revealed a non‐redundant physiological role for the three orthologs in rodents [27]. The availability of HeLa cell lines with complete absence of each GGA and of all three GGAs should allow more precise analysis of the role of the GGAs in these processes.

A striking observation noted with the *GGA123^−/−^* HeLa cells is the shift in CI‐MPR from peripheral punctae to the GM130‐positive compartment. Why is this the case? The GGAs bind directly to the CI‐MPR through an acidic‐dileucine motif located at the C terminus of the receptor [11, 12, 28, 29, 30]. This interaction is important in linking the CI‐MPR‐cargo complex with clathrin triskelia that assemble on the surface of the TGN, since the CI‐MPR itself does not bind directly to clathrin but does so via the intermediacy of the GGAs which function as clathrin adaptors ([11, 31]. We propose that in the absence of GGAs and GGA‐only CCV, AP‐1 assumes the exclusive role in mediating the nucleation of clathrin around the CI‐MPR, but this is insufficient in packaging all the cargo‐bound receptor into CCVs, resulting in a net accumulation of the CI‐MPR at the TGN. It is curious that the GM130‐positive compartment in the *GGA123^−/−^* triple‐knockout cells showed a broader than usual staining pattern for this Golgi marker. It has been reported that in addition to the conventional GGA‐containing CCVs that bud off the TGN, a population of highly pleiomorphic vesicular‐tubular carriers that contain both GGA1 and AP‐1, colocalized at the EM level, emanate from the TGN and move toward the cell periphery of MDCK cells [32]. Perhaps, this phenomenon contributes to the homeostatic maintenance of the TGN morphology of HeLa cells as well. In fact, Waguri *et al* demonstrated very similar tubulo‐vesicular structures containing AP‐1 and CFP‐CI‐MPR emanating from the TGN of HeLa cells [33]. It was not tested if GGAs were also present in these structures. It is possible that lack of GGA proteins in our *GGA123^−/−^* triple‐knockout cells impedes this process, thereby expanding the membrane surface of the TGN. It will be interesting to investigate if the GGAs contribute to delimiting of the TGN membrane.

In conclusion, we have shown that HeLa cells lacking all three GGAs are partially compromised in MPR‐mediated lysosomal enzyme sorting, with residual sorting presumably mediated by AP‐1. These various knockout cell lines can serve as useful tissue culture models for analyzing a number of additional pathways where the GGAs have been proposed to play an important role.

## Conflict of interest

The authors declare no conflict of interest.

## Author contributions

BD, LL, and SK conceived and designed the project. BD, LL, WSL, and BCJ acquired the data. BD, LL, and SK analyzed and interpreted the data; and BD and SK wrote the paper.

## Data Availability

Data will be available from the corresponding author upon reasonable request.
